# Xanthomatosis in a child following biliary atresia surgery: a case report

**DOI:** 10.1186/s13256-026-06457-2

**Published:** 2026-08-04

**Authors:** Rongkun Zhu, Yao Liu, Hanqi Zhang, Chunmei Bing, Qian Dong, Xiwei Hao

**Affiliations:** 1 Department of Pediatric Surgery, The Affiliated Hospital of Qingdao University, No. 16, Jiangsu Road, Shinan District, Qingdao, Shandong China; 2 Shandong Provincial Key Laboratory of Digital Medicine and Computer-Assisted Surgery, Qingdao, China

**Keywords:** Xanthomatosis, Biliary atresia, Reflux cholangitis, Hypercholesterolemia, Case report

## Abstract

**Background:**

Xanthomatosis as a metabolic disorder, is rare in children, especially in those who have undergone biliary atresia surgery.

**Case presentation:**

The patient was a 1-and-a-half-year-old boy, who was a male infant from China with East Asian ethnicity and diagnosed with Xanthomatosis after biliary atresia surgery. After systematic low fat, cholesterol-restricted diet and rational anti-inflammatory treatment for reflux cholangitis, the child's symptoms were controlled.

**Conclusions:**

Reflux cholangitis after biliary atresia may contribute to metabolic changes associated with xanthoma development.

## Introduction

Xanthomatosis is a metabolic disorder caused by the deposition of cholesterol and other lipids in the skin, tendons, and other tissues, manifesting as yellow-orange plaques or nodules in areas such as the tendons, eyelids, palms, and elbows. Xanthomas can be classified into multiple subtypes, including eruptive, nodular, tendinous, verrucous, and planar xanthomas, depending on their location and morphology [1].While xanthomas are most commonly associated with primary familial hypercholesterolemia, they can also occur secondary to liver damage, such as cholestatic cirrhosis[2].

Biliary atresia (BA) is a rare progressive inflammatory and fibrosclerotic disease of the biliary system and a leading cause of neonatal cholestasis[3, 4].Early treatment typically involves Kasai portoenterostomy or liver transplantation, but recurrent episodes of reflux cholangitis following surgery can result in persistent cholestasis, hepatocellular injury, and cirrhosis, increasing the risk of metabolic complications[5].

This report presents a rare case of Xanthomatosis in a 1-and-a-half-year-old East Asian Chinese boy after BA surgery, aiming to explore the potential associations and contributing factors underlying this uncommon manifestation.

## Case report

### Medical history and surgical treatment

The patient was a male infant of East Asian Chinese ethnicity who developed jaundice 2–3 days after birth. Due to family circumstances, the condition was not promptly addressed or treated. The jaundice persisted, accompanied by pale, clay-colored stools, and worsened within a week, leading to hospital admission.

By two months of age, the patient exhibited severe jaundice, raising suspicion of biliary atresia. The family declined a liver biopsy. Preoperative contrast-enhanced CT of the liver was performed, with dynamic hepatobiliary imaging at 5,10,15,20,25,and 30 minutes ,as well as at 1,3,and 24 hours after intravenous contrast administration. Between 5 and 30 minutes ,liver imaging gradually became clear, with normal position, size, and morphology, but without visualization of the bile ducts. By 30 minutes ,a small amount of contrast was excreted into the intestine. At 1 hour, the liver was clearly visualized, but the bile ducts remained undetectable. At 3 hours, the liver imaging remained clear, with no bile duct visualization, though contrast excretion into the intestine was observed. At 24 hours, both the liver and kidneys remained enhanced, with minimal contrast in the intestines. These findings suggested the possibility of extrahepatic obstructive (partial) jaundice.

After consultation with pediatric specialists to exclude neonatal hepatitis syndrome and discussion with the family, exploratory surgery was planned. On November 23,2022, an exploratory laparotomy was performed. Intraoperative findings revealed a hypoplastic, slender gallbladder. Upon incision, white secretions were observed, but no bile was present. Following the insertion of a scalp needle, contrast (iodipamide) was injected into the gallbladder, which flowed into the intestine but failed to visualize the intrahepatic or extrahepatic bile ducts, confirming a diagnosis of congenital type II biliary atresia. A Kasai portoenterostomy was performed as planned.

**Fig. 1:**
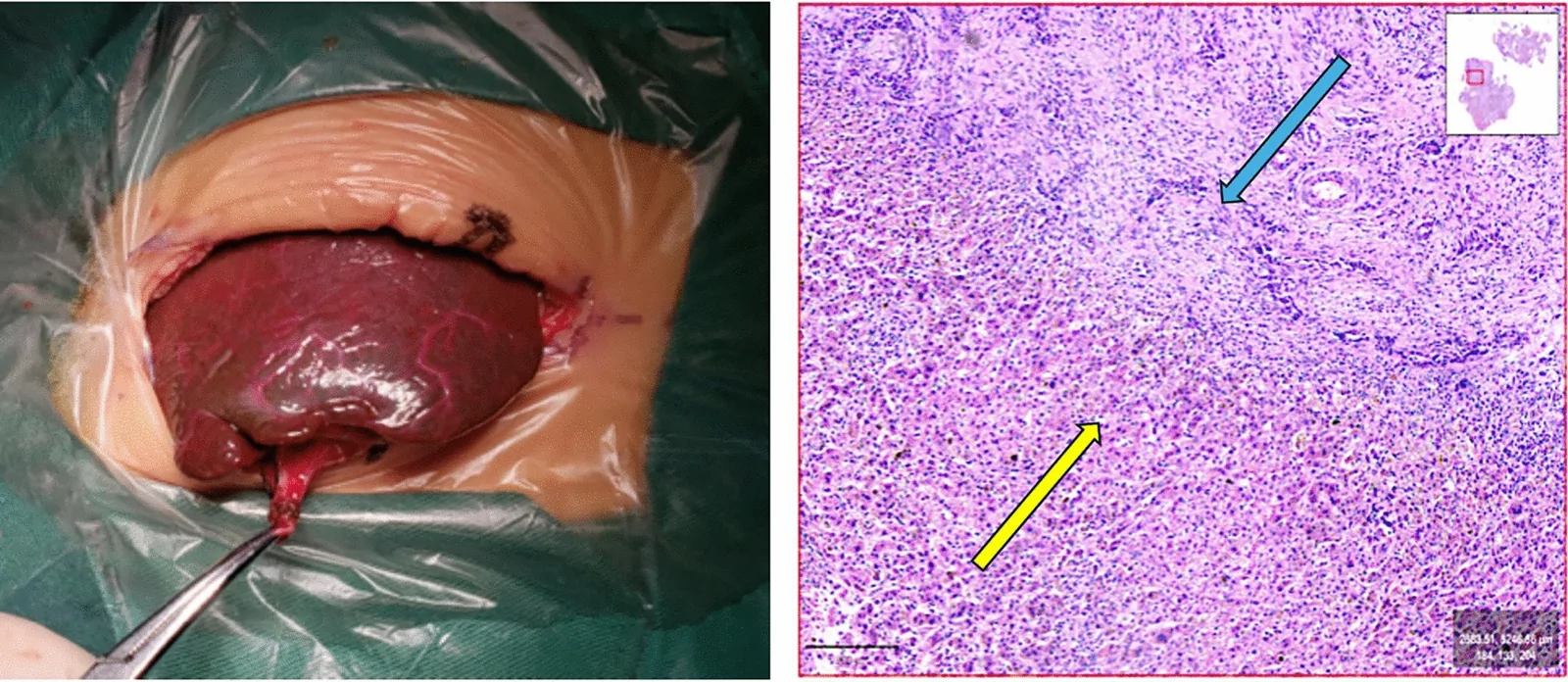
The left panel shows intraoperative image of the liver during Kasai surgery for the patient. The liver shows signs of abnormal texture, which may suggest the presence of cholestatic liver damage; the right panel shows a postoperative histopathological image (100 × magnification), with yellow arrows indicating cholestasis and blue arrows highlighting fibrotic changes

Postoperative histopathology revealed marked hepatocellular cholestasis, significant fibrous tissue proliferation in the portal area with extensive chronic inflammatory cell infiltration, and bile duct proliferation (Fig. 1).The patient recovered with systematic postoperative treatment and was discharged.

### Postoperative disease progression and treatment

Following discharge, the patient experienced five episodes of recurrent reflux cholangitis on December 29,2022 (postoperative day 36),January 7,2023 (day 45),January 27,2023 (day 65), February 14,2023 (day 83), and June 20,2023 (day 210).Each episode was managed conservatively at our hospital with hepatoprotective and choleretic therapy, including oral glycyrrhizin compound capsules for liver protection, ursodeoxycholic acid for bile flow improvement, and anti-inflammatory medications. (Ursodeoxycholic acid (UDCA) exerts its effect through two key mechanisms: displacing toxic bile acids and stimulating the secretion of bicarbonate-rich bile. This dual action results in improved intrahepatic bile flow and microcirculation.)

**Fig. 2:**
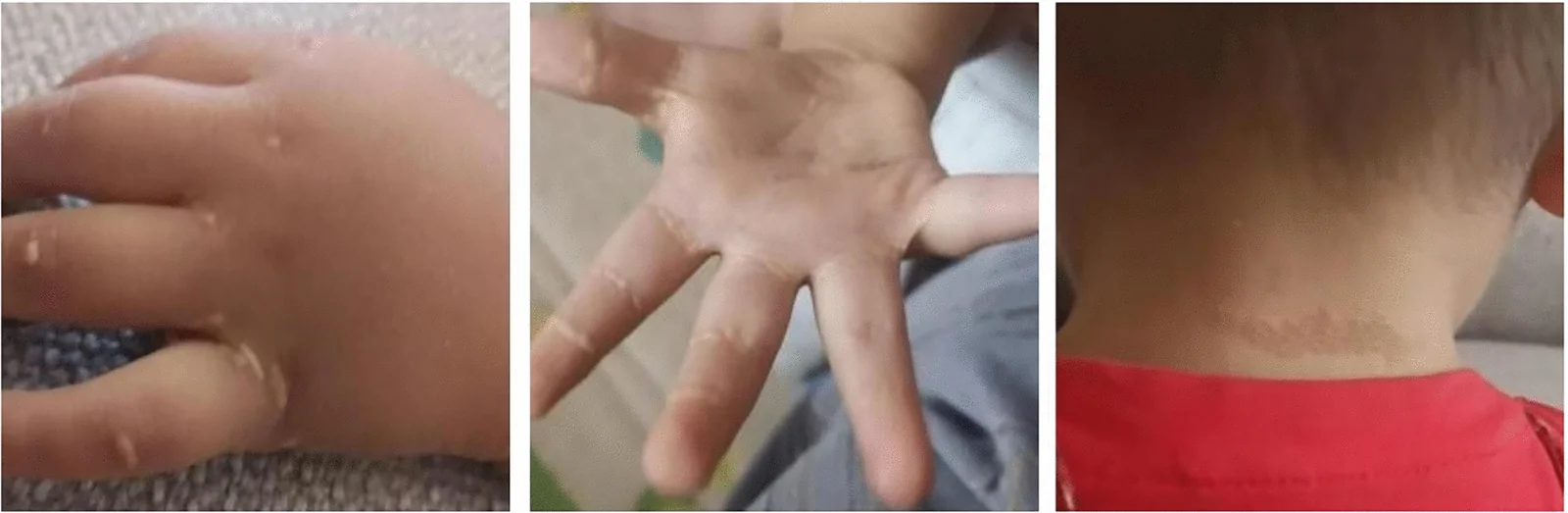
Pale yellow papules measuring 1–5 mm in diameter on the palms and dorsum of the hands, arranged in groups forming linear distributions along skin folds; clustered distribution on the upper back and neck

In December 2023,one year after surgery, the 1-and-a-half-year-old East Asian Chinese boy developed scattered pale yellow papules on the neck, upper back, wrists, dorsum of the hands, and palms (Fig. 2).Systemic findings included firm hepatomegaly (1 cm below the right costal margin) and splenomegaly (2 cm below the left costal margin).Nutritional assessment demonstrated significant growth retardation, with weight and height for age both below the 3rd percentile for corrected age. No ascites was detected. Laboratory tests revealed markedly elevated cholesterol and low-density lipoprotein (LDL) levels: total cholesterol 19.88 mmol/L (normal range:2.9–5.68 mmol/L),LDL 18.18 mmol/L (normal range: 0–3.37 mmol/L),and triglycerides 4.63 mmol/L (normal range: 0–1.7 mmol/L). Blood glucose and thyroid function were normal. Notably, both parents had normal lipid profiles. The patient was advised to follow a low fat, cholesterol-restricted diet.

### Laboratory assessment

**Fig. 3:**
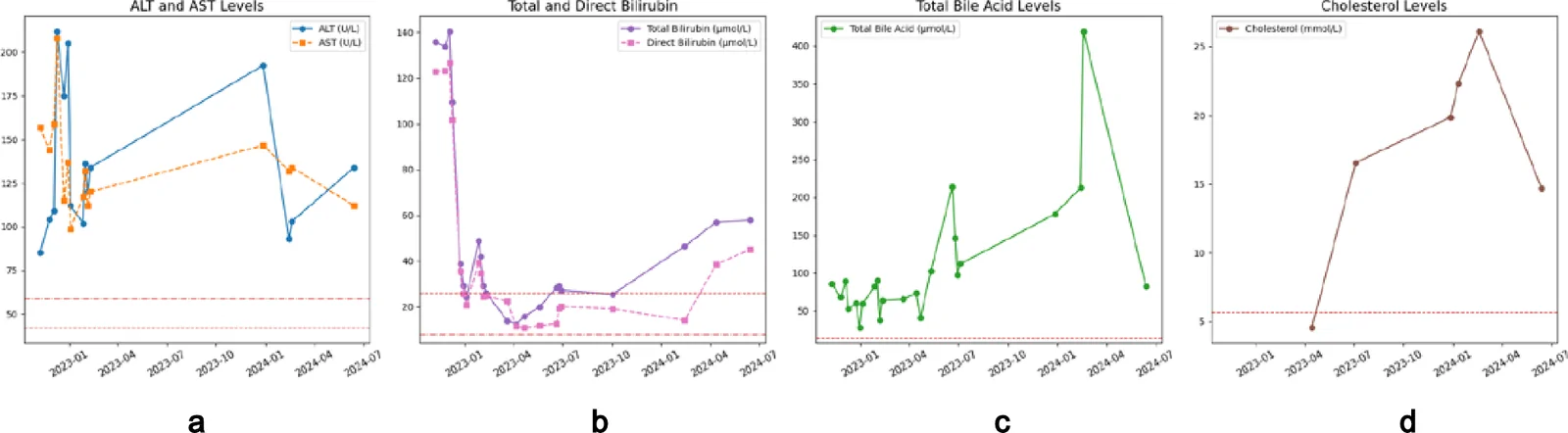
Serial changes in liver function tests over the disease course. **A** Alanine aminotransferase (ALT) and aspartate aminotransferase (AST) levels. **B** Total and direct bilirubin levels. **C** Total bile acid levels. **D** Serum cholesterol levels. Red dashed lines indicate the upper limits of the normal reference ranges for each parameter

The dynamic changes in liver function parameters, bilirubin levels, bile acid, and cholesterol levels after Kasai surgery reflect disease progression (Fig. 3).

- (a) Alanine aminotransferase (ALT) and aspartate aminotransferase (AST) were markedly elevated postoperatively, indicating hepatocellular injury. Levels gradually declined but remained above normal, suggesting persistent damage.
- (b) Total and direct bilirubin initially peaked and rapidly declined but did not normalize. Recurrent cholangitis led to a slight rebound, indicating impaired bile drainage.
- (c) Total bile acid remained elevated postoperatively and surged after June 2023, suggesting progressive cholestasis.
- (d) Cholesterol levels rose significantly postoperatively, peaking in October 2023 before slightly decreasing but remained abnormally high.

ALT/AST panel**:** Reference line at 40 U/L (upper normal limit for liver transaminases).

Total/direct bilirubin panel: Two reference lines at 17.1 μmol/L (total bilirubin) and 6.8 μmol/L (direct bilirubin).

Total bile acid panel**:** Reference line at 10 μmol/L (upper normal limit).

Cholesterol panel**:** Reference line at 5.2 mmol/L (upper normal limit for total cholesterol).

### Genetic sequencing

To investigate the cause of Xanthomatosis, peripheral blood samples were collected with parental consent for whole-exome sequencing (WES) and copy number variation (CNV) analysis using next-generation sequencing (NGS).

Genomic DNA was extracted, processed, and sequenced, covering 20,700 exonic regions (± 20 bp) and the full mitochondrial genome (16,569 bp).Data were aligned to the human reference genome hg19 (GRCh37), with quality metrics detailed in Table 1.

**Table 1:** Sequencing metrics

Patient			
Coverage of target genes	≥ 10X	≥ 20X	≥ 50X
	98.28%	98.11%	96.62%
Mean depth	121.77X		

A total of 1307 phenotype-related genes were screened, and variants were classified per 2015 ACMG guidelines. Large-fragment CNVs (≥ 2 consecutive exons) were analyzed, with quality-verified findings reported. Pathogenic or likely pathogenic autosomal recessive variants were confirmed via NGS and/or Sanger sequencing.

No significant variants, CNVs, or mitochondrial mutations were identified. A homozygous deletion in exon 3 of GHR was detected, autosomal dominant and autosomal recessive variations of the GHR gene are associated with the occurrence of familial high sitosterolemia. However, the deletion and loss of this variation in the non-repetitive box of the GHR gene leads to changes in protein length (PM4), and this variation has not been included in gnomAD v2.1.1 (PM2_Supporting), its clinical significance remains uncertain. The exact cause of Xanthomatosis in this East Asian Chinese patient is still unclear.

### Follow-up

During postoperative follow-up, the East Asian Chinese patient received ongoing conservative treatment, including choleretic and hepatoprotective therapy along with a low fat, cholesterol-restricted diet. Five episodes of reflux cholangitis occurred.

ALT, AST, total bilirubin, and direct bilirubin remained elevated but stabilized over time. Bile acid and cholesterol levels peaked at the onset of Xanthomatosis but gradually declined with treatment. The skin Xanthomas showed signs of improvement.

## Discussion

Xanthomatosis is a metabolic skin disorder caused by the deposition of cholesterol and other lipids in tissues such as the skin and tendons. Although rare in children, it can develop secondary to bile metabolism disorders [6].

In this case report, we analyzed the clinical course of a one-and-a-half-year-old East Asian Chinese male BA patient who developed Xanthomatosis after Kasai surgery. But it has several limitations. First, the diagnosis of Xanthomas was clinical and not confirmed by skin biopsy, which could have provided histopathological support. Second, we did not perform advanced lipid characterization, such as lipoprotein elecrtophoresis or apolipoprotein E genotyping, which might have further defined the specific subtype of dyslipidemia in these patients. We are limited in the conclusions that can be drawn from this single case. These limitations should guide more comprehensive assessments in future cases, the progression of Xanthomatosis in this patient may be linked to recurrent cholangitis, leading to bile stasis, hepatocellular injury, and subsequent cholesterol metabolism abnormalities.

BA is a severe pediatric hepatobiliary disorder characterized by cholestasis, hepatocellular injury, and progression to biliary cirrhosis [3]. It must be differentiated from neonatal hepatitis (NH), Alagille syndrome (ALGS), and congenital biliary anomalies. In this case, preoperative pale-colored stools, intraoperative findings of gallbladder hypoplasia, absent intra- and extrahepatic bile duct visualization on cholangiography, and postoperative pathology showing bile duct proliferation with fibrosis confirmed the diagnosis of BA while excluding NH. ALGS is typically associated with pulmonary artery stenosis, butterfly vertebrae, characteristic facial features, and renal anomalies, with liver biopsy revealing bile duct paucity; however, this patient exhibited no systemic abnormalities, and histopathology did not support ALGS [7]. Additionally, imaging ruled out congenital biliary anomalies, leading to a definitive BA diagnosis.

Kasai portoenterostomy remains the first-line surgical treatment for BA, with cholangitis as the most common postoperative complication, occurring in 30%-60% of cases, mostly within the first year. Early-onset cholangitis (within one month postoperatively) and recurrent cholangitis (≥ 3 episodes within six months) are associated with poor prognosis and liver transplantation [8–11]. This patient, operated on at less than two months of age, underwent an uneventful Kasai procedure. However, reflux cholangitis occurred 36 days postoperatively, with four additional episodes within six months. Laboratory findings showed improved liver function and a gradual decline in bilirubin levels, yet recurrent cholangitis likely prolonged bile retention, disrupting cholesterol metabolism.

To date, only two cases of Xanthomatosis following BA surgery have been reported, both identified as Xanthoma Striatum Palmare (XSP),a condition typically linked to type III hyperlipoproteinemia [12–15].

Genetic testing revealed a homozygous deletion in exon 3 of the GHR gene, while parental lipid profiles were normal. According to ACMG guidelines, this variant is classified as of uncertain clinical significance. Thus, the exact cause of Xanthomatosis in this patient remains unclear, though it is likely linked to cholesterol metabolism dysfunction due to cholestasis. The liver is the primary site of cholesterol production in the human body [16].

Following Kasai surgery, recurrent reflux cholangitis led to persistent cholestasis, impairing bile excretion into the intestine. The accumulated bile, rich in cytotoxic bile acids, directly damages hepatocytes and biliary epithelial cells while acting as pro-inflammatory mediators. These bile acids activate immune cells in the liver, inducing inflammation and cytokine release, disrupting the apical bicarbonate barrier of biliary epithelial cells, and promoting apoptosis. This process exposes pyruvate dehydrogenase complex E2 (PDC-E2) on the mitochondrial membrane, forming immune complexes with circulating autoantibodies, thereby exacerbating hepatocyte and biliary epithelial injury [17–19].

Additionally, bile deficiency alters gut microbiota composition, allowing bacteria and their metabolites to translocate to the liver via the portal vein, further aggravating inflammation and tissue damage. Chronic inflammatory stimuli activate hepatocytes and hepatic stellate cells (HSCs), transforming them into myofibroblasts (MFBs) that synthesize excessive collagen and extracellular matrix (ECM),driving hepatic fibrosis [20, 21].Cholestasis and fibrosis also damage sinusoidal endothelial cells and induce capillarization, leading to portal hypertension. To counteract this, the body develops collateral circulation, disrupting the enterohepatic circulation and exacerbating dysbiosis and bacterial translocation, creating a vicious cycle of inflammation and liver injury.

The disruption of enterohepatic bile acid circulation impairs cholesterol metabolism and excretion, reducing hepatic cholesterol clearance [22–24].Cholesterol is primarily eliminated through bile acid metabolism, but cholestasis decreases bile acid secretion, leading to cholesterol accumulation. Moreover, portal hypertension further affects hepatic cholesterol absorption, ultimately causing hypercholesterolemia and cholesterol deposition in the skin and tendons, forming xanthomas. Thus, in this patient, recurrent post-Kasai cholangitis led to cholestasis, gut microbiota dysbiosis, and liver fibrosis, all contributing to cholesterol metabolism dysfunction and Xanthomatosis. Although a GHR gene variant was present, no definitive evidence currently links it to Xanthomatosis.

## Conclusion

Although post-Kasai xanthomatosis is exceedingly rare, its occurrence highlights a severe dysmetabolic state in progressive chronic cholestasis. The pathogenesis likely involves a combination of cholestasis-induced liver fibrosis (e.g., secondary to reflux cholangitis) and individual metabolic susceptibility. This case demonstrates that a structured conservative approach—targeting bile flow, hepatoprotection, and gut-liver axis modulation—can effectively manage this condition, as evidenced by the regression of xanthomas and clinical stabilization. Consequently, our experience suggests that early detection and comprehensive treatment of hypercholesterolemia and cutaneous xanthomas should be integral to the long-term follow-up of post-Kasai patients to prevent additional morbidity.

## Data Availability

The raw sequencing data generated in this study are available in GSA ( https://ngdc.cncb.ac.cn/gsa) with the accession number HRA011264 or through the URL: https://bigd.big.ac.cn/gsa-human/browse/HRA011264. Other clinical data generated and/or analyzed during the current study are available from the corresponding author upon reasonable request.

## Abbreviations

BA: Biliary atresia
LDL: Low-density lipoprotein
ALT: Alanine aminotransferase
AST: Aspartate aminotransferase
WES: Whole-exome sequencing
CNV: Copy number variation
NGS: Next-generation sequencing
NH: Neonatal hepatitis
ALGS: Alagille syndrome
XSP: Xanthoma striatum palmare
PDC-E2: Pyruvate dehydrogenase complex E2
HSCs: Hepatic stellate cells
MFBs: Myofibroblasts
ECM: Extracellular matrix
UDCA: Ursodeoxycholic acid
